# Reporting guidelines in global health research

**DOI:** 10.7189/jogh.06.020101

**Published:** 2016-12

**Authors:** Ana Marušić, Harry Campbell

**Affiliations:** Editors in Chief, Journal of Global Health

Reporting guidelines have become important tools in health research. They improve the accuracy, completeness and transparency of reporting most important aspects of research studies [1]. This is particularly important not only for accurate evaluation of the methodological quality of research and validity of the results, but also increases the quality of evidence synthesis from health research for practical application [2]. Research that is not clearly reported leads to waste in research, distorts existing evidence and compromises the replication of research findings. It has been well demonstrated that the endorsement of the CONSORT – the first reporting guideline, developed for randomized controlled trials – increases the completeness of completeness of trial reporting in medical journals [2].

The central part of a reporting guideline is a checklist which lists minimum information that must be provided in a published article. Checklists made their way into health research reporting from industry, where they, just as in medicine, serve as quality and safety assurance of processes and products, particularly those involving high risk [3]. A good example of a medical checklist is the WHO Surgical Safety Checklist, which clearly demonstrated a highly significant reduction in complication or death rates after surgery [4,5].

There are currently more than 300 reporting guidelines for different study designs and health research disciplines –the most comprehensive source of information is the EQUATOR Network [6]. The *Journal of Global Health* (*JOGH*) requires from its authors to follow relevant reporting guidelines when submitting their manuscript [7].

We would like to draw your attention to newly published reporting guidelines that are relevant for global health.

The reporting guideline “Strengthening the Reporting of Observational Studies in Epidemiology for Newborn Infection” or STROBE-NI is an extension of the STROBE statement for neonatal infection research. It was recently published in *Lancet Infectious Diseases* [8] and is also available at the EQUATOR Network site [9]. Neonatal infections account for about one quarter of the 2.8 million neonatal deaths globally each year [10] and there is an urgent need for research investment to define new approaches to tackle this problem. As noted by the authors, the guideline seeks to “optimise reliability, clarity and comparability of scientific reporting of neonatal infections in observational studies and relevant trials, especially aetiological (bacterial, viral and fungal) data, to maximise their utility and impact.” This fills an important gap in this important topic and will complement existing guidelines related to child health trials and systematic reviews and meta-analyses. The SPRING checklist follows the STROBE format and focuses on key issues important to the design and reporting of neonatal infection research. Importantly, the SPRING group included substantial expertise from researchers from low and middle income countries to ensure they are relevant to these high burden settings.

The Guidelines for Accurate and Transparent Health Estimates Reporting or GATHER statement was first published in the *Lancet* [11] and *PLoS Medicine* [12]. It is also available at the site of the GATHER statement [13], as well as the EQUATOR Network [14]. Due to very incomplete data in most countries globally statistical or mathematical models are often used to estimate key health indicators. These are then used to inform global and national decision-making and priority setting. It is important, therefore, that we can have confidence in the estimates and are able to understand how they were derived and what the uncertainty bounds are surrounding the estimates. In order to promote accurate interpretation and responsible use of these data, World Health Organization (WHO) convened a number of expert consultations to prepare a set of standard reporting guidelines – Guidelines for Accurate and Transparent Health Estimates Reporting (GATHER) [11,12]. The guideline comprises a checklist of 18 essential items for best reporting practice. An important aspect of GATHER is that the data underlying the modeled estimates are made accessible online. Given the importance of these principles in promoting best practice in studies generating data to underpin evidence-based policy making, we strongly support this initiative. We hope that medical and public health journals will ensure that burden of disease studies that fall within the ambit of these guidelines will be required to implement these guidelines before accepted for publication.

Finally, the newest reporting guideline addresses the translation of evidence to practice by providing a reporting guideline for health care practice guidelines. The guideline is published in the *Annals of Internal Medicine* [15], and is also available at the EQUATOR Network [16] and the web-site of the RIGHT statement [17]. This guideline is the results of a working group which addressed the problem of often poor reporting of health care practice guidelines despite their importance and popularity as a tool to improve the quality of health care. The reporting guideline was developed as a joint effort from expert and consumer representatives, including internationally recognized organizations such as Guidelines International Network, Cochrane Collaboration, AGREE Collaboration, GRADE working group, International Society for Evidence-Based Health Care, EQUATOR Network, and National Guideline Clearinghouse [15,17]. The guideline comprises a checklist with 22 items and a flow diagram, as well as an explanation and elaboration paper.

In addition to the requirement to use a relevant reporting guideline when submitting their manuscripts to the *Journal of Global Health*, we encourage our authors and readers with an interest in the fields covered by the three reporting guidelines to help disseminate the guidelines and promote their use. Also, contact guideline developers with comments and suggestions about improving and further developing these reporting guidelines.
